# Societies

**Published:** 1867-08

**Authors:** 


### SOCIETIES.

It is often suggested to us that our list of Societies is not correct. It is not an easy matter to keep it correct without the co-operation of the officers of societies. We trust that hereafter, when there is any change made in any of our societies, either in respect to officers, time or place of meeting, that we will be at once notified of the fact, that the proper correction may be made. The table is interesting to all only in proportion to its accuracy.

